# An open-source molecular diagnostic platform approach for outbreak and epidemic preparedness

**DOI:** 10.4102/ajlm.v9i2.1017

**Published:** 2020-09-28

**Authors:** Devy M. Emperador, Laura T. Mazzola, Cassandra Kelly-Cirino

**Affiliations:** 1 Foundation for Innovative and New Diagnostics, Geneva, Switzerland; 2 Foundation for Innovative and New Diagnostics, San Francisco, California, United States

**Keywords:** diagnostics, development, outbreak, preparedness, infectious disease, test development

## Abstract

**Background:**

Diagnostic development for outbreak pathogens has typically followed a disease-specific reactive rather than proactive response. Given the diversity of outbreak pathogens, particularly those prioritised by the World Health Organization Research and Development Blueprint, a more flexible and proactive approach to epidemic preparedness is needed to expand access to critical molecular diagnostic tests in peripheral and resource-constrained deployment settings.

**Objective:**

New and more sustainable directives are needed to spur the development of high-quality products, particularly for epidemics more often found in low- and middle-income countries. To leverage and de-risk the development process, we present the benefits and challenges of an open-source business model for co-development of molecular diagnostic tests for decentralised settings.

**Methods:**

We identify key outbreak pathogens that are available only for testing in high infrastructure laboratories and compare in-country installed base platforms that could be leveraged for menu expansion. Key strengths and challenges for development are highlighted for both platform and assay developers, with discussion of how to leverage and de-risk the process through an open-source development model.

**Results:**

Depending on the specific partner strengths, options for partnership roles are presented. The proposed open-source business model addresses the particular challenges in the detection of outbreak- and epidemic-prone pathogens in low- and middle-income countries, reduces development and deployment risks to support outbreak response, strengthens diagnostic capacity and creates a viable market for product developers.

**Conclusion:**

We hope this model for a collaborative and open-source approach for molecular diagnostics serves to encourage stakeholders to consider co-development partnerships to improve outbreak preparedness and epidemic/pandemic response.

## Introduction

Diagnostics are a fundamental component of a successful outbreak response by enabling surveillance and early detection, rapid and appropriate patient management, and objective evaluation of outbreak containment, in addition to facilitating therapeutic and vaccine development.^1,2,3,4^ For outbreak and epidemic-prone pathogens, diagnostic development has typically followed a disease-specific reactive approach. Faced with significant mortality and morbidity during the 2013–2016 Ebola virus disease and 2015–2017 Zika virus epidemics, substantial global funding was made available, resulting in the rapid development of new diagnostics, therapeutics and vaccines.^5,6,7,8^ However, only a handful of the diagnostic tests survived to become commercial products, and few found a sustainable market once the epidemics were declared contained and public funding was diverted elsewhere.^5,9,10^

A shift toward a more proactive and nuanced development strategy for outbreaks and epidemics is needed, especially to support diagnostic capacity in low- and middle-income countries (LMICs). Some epidemic-prone diseases are endemic in certain regions, requiring diagnostics that can differentiate active disease from background exposure. For example, Lassa fever affects many countries in West Africa, with resurging high rates of cases during the dry season.^11,12,13^ Likewise, some emergent diseases share overlapping geographies and similar clinical symptoms (e.g. Lassa fever and Ebola virus disease, Middle East Respiratory Syndrome coronavirus and influenza), an additional challenge for rapid and definitive diagnosis.^2,14^ Rapid pathogen identification can be critical when early detection can direct lifesaving treatment and intervention, such as antibiotics for confirmed bacterial infections^15^ or vector control vaccination campaigns during a yellow fever outbreak.^2^

There are common challenges to the deployment of diagnostic tests during outbreaks.^16^ Early detection and contact tracing at the first stages of an outbreak are critical to rapid containment. Similarly, a rapid test turn-around time enables patients to be triaged both quickly and appropriately. In settings of endemic diseases with similar clinical indications, it will be invaluable to rapidly differentiate and identify the pathogen responsible for the outbreak. This kind of dynamic outbreak response demands access to diagnostics across a wide range of infrastructure, from reference laboratories to near-patient (NPT) hospital laboratories to point-of-care (POC) clinics and community testing.

In particular, the priority pathogens identified by the World Health Organization Research and Development Blueprint^17^ present a challenge for diagnostic development for epidemic preparedness by any single developer, as the pathogens cover a wide range in clinical presentation, transmission mode, specimen type, lineage diversity, geography and outbreak frequency. A more flexible approach is needed to rapidly develop or adapt diagnostic tests for a wide variety of pathogens, which can be implemented in outbreak-appropriate deployment settings.^18,19,20^

## Molecular diagnostics for epidemic preparedness

Molecular diagnostics (MDx) plays an important role in disease diagnosis (detection and confirmation of active infection), case management and surveillance, as well as a supporting role in therapeutic and vaccine trials. Due to the processing complexity and risks of contamination, MDx testing capacity is traditionally available only at reference-level or high-resourced laboratories, which can significantly delay patient diagnosis and intervention during an outbreak when specimen transport is a challenge. Molecular diagnostics testing is particularly useful at peripheral health facilities, where sick patients first present, to allow for pathogen detection. However, peripheral settings typically have limited resources for laboratory testing: NPT settings describe hospital and clinic-associated laboratories or mobile units, which support a minimal level of laboratory infrastructure and personnel; POC settings typically lack any laboratory infrastructure, including bedside, primary care and community-based testing.^21^

Near-patient/point-of-care MDx platforms have been designed specifically to meet the requirements for rapid pathogen detection in low-infrastructure settings,^22,23,24,25,26^ with a compact and automated design for a simplified ‘sample in, result out’ processing.^18,19,27,28^ Near-patient/point-of-care MDx target product profiles have been described in detail.^29,30,31,32^ Successful implementation has been demonstrated in decentralised laboratories in LMICs for diseases including HIV, tuberculosis, Zika virus, Ebola virus disease and influenza,^22,23,24,33,34,35,36^ suggesting that a broader menu of assays could be developed for these NPT/POC platforms for outbreak detection.

### Outbreak pathogen molecular diagnostics opportunities

Table 1 presents a list of outbreak pathogens identified as high priority pathogens for medical countermeasures due to their high epidemic and pandemic potential.^37,38,39^ Most of these diseases have MDx tests (reagent kits) that are commercially available, but appropriate only for use in high infrastructure research laboratories. In some cases, the MDx test has not been validated clinically or approved for diagnostic use (i.e. research-use-only kit), while in other cases, particularly for newer pathogens, MDx tests are available only as published protocols from trusted reference laboratories.^17,38,40^ There is potential for available laboratory kits to be re-engineered for NPT/POC testing, including kits for viral haemorrhagic pathogens^41,42,43,44^, arboviral diseases,^45,46,47,48^ respiratory pathogens^49,50,51^ and biothreat pathogens (including Disease X).^39,52,53^

**TABLE 1:** Availability of molecular diagnostic tests for select outbreak pathogens.

Pathogen	MDx test commercially available	Regulatory approval†	POC MDx option
Ebola virus disease (EBOV)	Y	Y	Y
Marburg virus (MARV)	Y	Y	Y
Lassa virus (LASV)	Y	Y	–
Crimean-Congo haemorrhagic fever virus (CCHF)	Y	–	–
Nipah virus (NiV) and related henipavirus‡	–	–	–
Middle East Respiratory Syndrome Coronavirus (MERS-CoV)	Y	Y	–
Severe acute respiratory syndrome virus (SARS-CoV)	Y	Y	–
2019 novel Coronavirus (SARS-CoV-2)	Y	Y	Y
Influenza virus (A/H1N1/2009)	Y	Y	–
Zika virus (ZIKV)	Y	Y	Y
Chikungunya virus (CHIKV)	Y	Y	–
Yellow Fever virus (YFV)	Y	Y	–
Rift Valley Fever virus (RVFV)	Y	Y	–
Monkeypox virus (MPV)‡	–	–	–
Vibrio cholerae	Y	Y	–
Neisseria meningitidis	Y	Y	–
Yersinia pestis	Y	–	–

MDx, molecular diagnostics; POC, point-of-care.

† , Approved by stringent regulatory authority, for example, Conformitè Europëenne marking, United States Food and Drug Administration approval, etc. In many cases, clinical validation is limited to developer self-reported data available in product insert.

‡ , Published protocol(s) for laboratory assay, research-use-only.

## New diagnostic development approach

### Traditional assay development model

Tests for novel diseases are typically developed in academic or national reference laboratories which have the expertise, infrastructure and resources for rapid development and validation of MDx tests. These ‘in-house’ or laboratory-developed MDx tests are published and distributed as protocols from an internationally trusted source.^44,54,55,56^ However, the test methods require a high level of expertise and are generally limited in deployment to other reference-level laboratories. Commercial products can enable broader deployment, where the MDx test is manufactured as quality-assured kits that contain all of the necessary pre-measured test reagents.

In the traditional development model, a commercial platform developer builds a portfolio of proprietary assays, using a cartridge specific to the developer’s instrument platform. The instrument, cartridge, reagents and consumables are manufactured by the platform developer and supported as a ‘sole source’ provision. Commercial development requires significant financial resources from the developer, with the cost of test development, manufacturing, clinical validation and regulatory approval generally considered to be an investment toward sustainable sales. Companies require some profit margin in order to sustain their business and further develop test menus. When the anticipated markets are small, such as for disease with limited or intermittent outbreaks, or where prices are constrained to the cost of goods (as is typical with global health/LMIC markets), then there is little incentive for commercial development. In many resource-constrained settings, alternative test options are not affordable or simply not available. To support rapid menu expansion during new or repeated outbreaks, a more flexible concept for outbreak pathogen test development is needed.

### Open-source diagnostic model

In LMIC settings, leveraging the existing diagnostic infrastructure is key to expanding the capacity for disease management and outbreak preparedness. Rather than requiring repeated *de novo* investment, existing platforms could be utilised for test menu expansion, building on the existing human resources, laboratory testing capacity and supply chains. A similar approach has seen success in the integration of tuberculosis- and HIV-testing at peripheral health centres.^57^ For outbreak response, more flexible initiatives are needed to support rapid test development and deployment scenarios.

Here we describe a new open-source business model for MDx test development using existing platforms for NPT/POC diagnostic testing. As a key innovation, the open-source development model introduces an open-architecture cartridge designed to facilitate rapid development of tests by outside assay developers, where the platform developer provides an open, sterile cartridge that can be used by assay developers (academic or commercial) to rapidly prototype MDx tests for the existing platform. This development model leverages the strength of each developer, namely the available install base of a platform within relevant LMICs and the availability of reagent kits for the detection of outbreak pathogens, albeit for high infrastructure laboratory use. As noted in Table 2, this open-source development model can serve to decouple some of the risks for new test deployment.

**TABLE 2:** Co-development leverage and risk reduction.

Developer	Strengths	Key risks
Platform developer	Core expertise: automated NPT/POC diagnostic platform with validated assays Installed base, customers in key LMIC settings LMIC-based support and supply chains	Core expertise: assay development with broad test menu including outbreak pathogens Low(er) costs and rapid turn-around for new assay development Low barrier for kit/reagent manufacturing, option for ‘on demand’
Assay developer	High development cost for integrated assay cartridges Manufacturing process less flexible Unused cartridges expire, expensive Companies may have limited capacity to support broad test menu	MDx kits intended for high infrastructure laboratories, not appropriate for resource-constrained settings Standard reagent kit requires high level of user expertise, manual processing

NPT/POC, near-patient, point-of-care; LMIC, lower middle-income country; MDx, molecular diagnostic.

This open-source model is intended to catalyse a more rapid approach to the development of diagnostic tests for decentralised or low-infrastructure settings, and to provide a more flexible approach to manufacturing tests for novel and endemic pathogens for LMICs through co-development (Table 3). Variations on the co-development partnership include collaborative research, test kit sub-contract manufacturing, and licensing or acquisition. These variations each require specific research and development and supply agreements, as well as marketing and product support agreements between the partners. In some cases, a highly successful development partnership could lead to downstream acquisition. Even in cases with limited sales, the ability to rapidly develop and deploy NPT/POC tests for novel pathogens is valuable in improving local outbreak response.

**TABLE 3:** Co-development roles for open-source model.

Category	Platform developer	Assay developer
Platform development	Manufactures instrument and high-volume sealed assay cartridges.	None.
Assay development	In-house assay development for big-market tests with integrated cartridges. Option for in-licensing for test reagents.	Develops test reagents for open cartridge for use on platform.
Assay validation	Validation and registration for sealed cartridges pre-loaded with reagents.	Validation for reagent kit.
Manufacturing	Source for instrument, sealed assay cartridges, sterile open cartridges.	Source for assay reagents with ‘on demand’ production capacity.
Product support	Platform developer provides all instrument and assay support for menu portfolio. Platform developer supports instrumentation and supply chain. Supply agreement between platform developer and assay developer.	Assay developer supports assay and supply chain. Supply agreement between platform developer and assay developer.
Marketing and deployment options	Assay developer provides contract research with licensing agreement to platform developer. Assay developer is OEM source for cartridge-ready reagent kits. Assay developer is independent source for reagent kits and cartridges, supply and co-marketing agreement with platform developer.	

OEM, original equipment manufacturer.

### Model benefits and challenges

An open-source model for diagnostics can provide a more flexible and cost-effective approach to MDx deployment in peripheral settings, provided that commercial developers can find mutual benefit in a strategic partnership rather than traditional competition. The co-development model outlined above is intended to leverage the differential strengths between assay and platform developers by decoupling the investment risk for test menu expansion to include pathogens that may have limited but critically important deployment.

By leveraging existing assays and NPT/POC infrastructure, new tests can be rapidly re-engineered with a more cost-effective and ‘on demand’ approach for manufacturing. For the platform developer, a comprehensive assay menu expands the user base, which may provide more resilient and sustainable market volumes for diagnostic manufacturing. For the assay developer, their existing assay portfolio is expanded to include testing across a broad range of settings.^58,59,60,61^ Designed to utilise existing LMIC resources, an acknowledged potential disadvantage of this business model is reduced competition in favour of entrenched player market stability and product availability. Novel molecular platforms must be evaluated, both for their potential implementation in LMICs, as well as their openness to work with partners in developing assays, particularly for outbreak-prone diseases.

To support this new development model, innovative partnerships and finance solutions are needed, especially for proactive development and commercial viability. Here, the role of product development partners can serve as both matchmaker and co-investor to incentivise co-development partnerships.^62^ The open-source model is currently being piloted by the Foundation for Innovative and New Diagnostics to partner diagnostic assay and platform developers in support of outbreak preparedness in the near term (2–5 years).^58^ Similarly, this development model would also benefit from support from the global health community toward sustainable market commitments, pooled procurement mechanisms, and funding for test stockpiling to establish more sustainable supply chains and support long term commitments to diagnostic manufacturers.^16^ Some of these mechanisms are in place to address similar challenges in the vaccines sector and should be explored and adapted for diagnostics.^63^

### Conclusion

Despite the influx of interest at the onset of a new outbreak, it is more often the case that research and development costs for new diagnostics may be too high relative to market size to provide a sufficient incentive for product development, and sustained support can be particularly challenging, for pathogens that present seasonal or sporadic markets. Alternative business models for diagnostic development could help reduce the high costs associated with bringing a new pathogen test to market. An open-source development partnership model can combine the strengths of commercial developers to reduce development the cost and time to market, while supporting existing supply chain infrastructure, training and proficiency.^29,32^

Certainly, there are additional upstream and downstream challenges for pathogen test development. For new or poorly understood outbreaks, there are uncertainties in pathogen identification, epidemiology and disease kinetics. Newly developed tests require additional resources for clinical validation, for which well-characterised specimens and calibration standards may be difficult to obtain.^16^ Commercial products also require international and/or in-country regulatory approval, though this may be a lower hurdle for assays that were previously validated as laboratory tests. Here, Product Development Partnerships can also serve as matchmakers to allow for sample sharing between research sites and developers with promising technologies, as well as to leverage in-country experience to support evaluation studies in LMICs that meet the requirements for regulatory approval.

The availability of appropriate diagnostics is a key feature for outbreak and epidemic preparedness. Early diagnosis of patients at initial point-of-care will enable timely patient and population interventions and support national surveillance systems for emerging and re-emerging diseases. Collaborative efforts are needed to develop NPT/POC diagnostics for all World Health Organization priority pathogens, to ensure access to high-quality diagnostics where testing is needed and to enable innovative funding mechanisms to support proactive development and market sustainability. We hope this article serves to encourage all diagnostic stakeholders (industry, academia, non-for-profit, non-governmental-organisations, governments) to foster partnerships in development and consider the open-source model early in the development of new diagnostics.

Lessons learned

- Diagnostic tests are critically needed for new and recurring outbreak response in peripheral and LMIC-appropriate settings.
- Leveraging the existing diagnostic infrastructure and expertise will be key to expanding disease management and outbreak preparedness.
- New business models such as open-source co-development partnerships can enable more flexible and proactive support for outbreak pathogen diagnostics.
